# Effectiveness of Sprayed Bioactive Fruit Extracts in Counteracting Protein Oxidation in Lamb Cutlets Subjected to a High-Oxygen MAP

**DOI:** 10.3390/foods9111715

**Published:** 2020-11-22

**Authors:** D. Morcuende, C. Vallejo-Torres, S. Ventanas, S. L. Martínez, S. C. Ruiz, M. Estévez

**Affiliations:** 1 IPROCAR Research Institute, Food Technology, University of Extremadura, 10003 Cáceres, Spain; demorcuen@unex.es (D.M.); sanvenca@unex.es (S.V.); 2 Food Engineering School, Technical State University of Quevedo, 120305 Quevedo, Ecuador; vallejot146@yahoo.es; 3 Meat Quality Laboratory, Santiago del Estero National University, G4200 Santiago del Estero, Argentina; sandraluz08@gmail.com; 4 National Institute of Agricultural Technology (INTA), 16003 Santiago del Estero, Argentina; ruiz.silvana@inta.gob.ar

**Keywords:** fruit extract, bioactive compounds, consumer perception, protein carbonylation, thiol oxidation, texture

## Abstract

High-oxygen packaging atmosphere (High-Ox-MAP) promotes meat protein oxidation and leads to texture deterioration. This study was conceived to assess the extent to which sprayed fruit extracts could inhibit the oxidative damage to proteins in lamb cutlets subjected to High-Ox-MAP (10 days/4 °C) and subsequent roasting (10 min/180 °C). Extracts from oaknut (*Quercus ilex* subsp. ballota; QI), rose hips (*Rosa canina* L.; RC), common hawthorn (*Crataegus monogyna* Jacq.; CM) and *strawberry tree* (*Arbutus unedo* L.; AU) were characterized for bioactive compounds (phenolic subclasses, tocopherols and ascorbic acid) and in vitro bioactivities. While the four fruits showed relevant antioxidant potential, CM had the highest phenolics and tocopherol content and that was reflected in efficient antiradical activity. The in vitro activity of this fruit to inhibit meat protein oxidation was, however, lower than that displayed by the other fruits. Taking the results altogether, CM was also found to be most efficient in protecting lamb cutlets from lipid oxidation. All fruits were able to inhibit thiols oxidation except RC, which seemed to reduce protein thiols. Among fruits, QI was the most efficient in protecting lamb cutlets against protein carbonylation as a plausible involvement of ellagitannins. The inhibition of protein oxidation by QI was reflected in significantly lower instrumental hardness in cooked lamb cutlets. Spraying lamb cutlets with extracts from QI, RC and CM improved consumers’ purchase intention after chilled storage. This antioxidant strategy seems to be a feasible and efficient solution to the pro-oxidative effects caused by High-Ox-MAP in red meat.

## 1. Introduction

Upon slaughter, the impairment of physiological processes in post-mortem muscle affects the ability of cells to counteract the pro-oxidant action of reactive oxygen species (ROS) [1]. As a result, muscle proteins are oxidized during aging and further on, during storage, processing and culinary treatment [2]. The oxidative degradation of thiols and the accretion of protein carbonyls and crosslinks cause severe physico-chemical changes in meat proteins with relevant consequences in terms of meat quality [2]. Protein oxidation is recognized to be responsible for the loss of nutritional value of meat owing to the depletion of essential amino acids and the impaired digestibility of oxidized meat proteins [2,3]. The intake of oxidized proteins and amino acids has also been found to induce postprandial oxidative stress in the gastrointestinal tract and in particular internal organs such as the liver, pancreas and brain [4]. Recent in vitro studies revealed some of the molecular mechanisms implicated in the toxicological effects of food-occurring oxidized amino acids in human intestinal and mouse pancreatic cells [5,6] In terms of sensory properties, numerous scientific pieces of evidence support the implication of protein oxidation in meat toughness [7,8,9]. This effect is particularly remarkable in red meat subjected to a high-oxygen modified atmosphere packaging (High-Ox-MAP) [10]. The necessity of guaranteeing the formation of oxymyoglobin and the display of a desirable bright red color gets into conflict with the onset of oxidative reactions, with the latter being significantly stimulated in meat stored in High-Ox-MAP [11,12]. Increasing oxygen concentration in MAP leads to increased lipid and protein oxidation rates and that is reflected in altered texture traits and low consumer acceptability [11,13].

A number of antioxidant strategies have been proposed to counteract the manifold and relevant consequences of protein oxidation on meat quality [14]. Phytochemicals naturally present in the plant kingdom (i.e., phenolics, tocopherols and terpenes) have concentrated considerable attention owing to their assorted bioactivities and are well accepted among consumers [15]. The addition of such antioxidant compounds to intact meat products requires the application of innovative procedures such as marinating [16], spraying [17], injection [18] or application of edible coatings [19] or active packaging systems [20]. The implementation of some of the aforementioned antioxidant strategies has led to benefits in terms of protection against lipid oxidation and inhibition of off-flavors. The protection against protein oxidation is more challenging since the effect of bioactive compounds on meat proteins depends on various and complex molecular mechanisms of interaction and hence, proper identification of such bioactive compounds is essential [21]. This is particularly relevant, as particular phenolic compounds have been reported to exert pro-oxidant actions towards meat proteins [14]. Therefore, only by understanding the molecular basis of the redox effects of plant phenolics on meat proteins, efficient and consistent antioxidant strategies against meat protein oxidation could be achieved.

The present study was carried out in two phases. In the first, several fruits, namely, oaknut (*Quercus ilex* subsp. *ballota*), rose hips (*Rosa canina* L.), common hawthorn (*Crataegus monogyna* Jacq.) and strawberry tree (*Arbutus unedo* L.) were characterized for their content in bioactive compounds and in vitro bioactivities. Subsequently, extracts of the aforementioned fruits, rich in bioactive compounds, were sprayed on lamb cutlets and tested for their ability to protect meat proteins against oxidation while packed and stored in High-Ox-MAP. In addition to oxidation markers, fresh, refrigerated and cooked lamb cutlets were also evaluated for quality traits (instrumental texture) and consumers’ purchasing decisions based on visual perception.

## 2. Materials and Methods

### 2.1. Chemicals and Raw Material

All chemicals and reagents were acquired from Panreac (Panreac Química, S. A., Barcelona, Spain), Merck (Merk, Darmstadt, Germany), Extrasynthese (Genay, France) and Sigma Chemicals (Sigma-Aldrich, Steinheim, Germany). The solvents employed for bioactive extraction from fruits were well suited for industrial food use. Fruits were collected at full ripeness in the Cáceres region (Spain) and subsequently lyophilized and frozen at −80 °C until used. Thirty Merino lambs raised outdoors in Cáceres province (Spain) were slaughtered at 25 kg of live weight (approx. 90 days of age) at a slaughterhouse in Cáceres (Spain). Carcasses were allowed to cool down for 24 h, after which racks were obtained. External fat was trimmed from racks, which were successively divided into cutlets of 70 g weight, 1.2 cm thickness, and approx. 12 cm^2^ of lean muscle area.

### 2.2. Extraction and Characterization of Fruit Extracts

For the preparation of fruit extracts, 100 g of sample including peel (except oaknut, which peel was removed) and pulp were cut into pieces, lyophilized and frozen at −80 °C. Twenty grams of lyophilized samples were finely ground and homogenized for 3 min with 10 volumes of 60% (rose hips and strawberry tree) or 80% food-grade acetone (oaknut and common hawthorn). The extraction procedure was optimized in a preliminary study to maximize the concentration of bioactive compounds. The homogenates were centrifuged at 2600× *g* for 10 min at 6 °C. The supernatants were collected and the residue was re-extracted once more following the procedure previously described. The two supernatants were combined and subsequently dried using a rotary evaporator at 40 °C, dispensed in 25 mL volumetric flasks, brought to volume with distilled water and immediately transferred to the laboratory for characterization.

The total phenolic content of each extract was determined following the Folin–Ciocalteu method [22] with slight changes. A diluted aliquot of each extract (200 μL; 1:200 *v*/*v* in water) was added to of diluted Folin–Ciocalteu’s reagent (1000 μL, 1:10 *v*/*v*) and 7.5% (*w*/*v*) sodium carbonate (800 μL). The combination was thoroughly mixed and allowed to stand in the dark for 45 min and at room temperature. Finally, absorbance was measured at 765 nm (Hitachi spectrophotometer, Tokyo, Japan). A standard curve of gallic acid was used to calculate the concentration of total phenolics. Results are shown as mg gallic acid equivalents (GAE) per 100 g of dry matter.

Analysis of tocopherols was carried out in accordance with the procedure reported by Rodriguez-Carpena et al. [23]. One milliliter of fruit extracts was redissolved with isopropanol (1:10, *v*/*v*) prior to analysis by a Shimadzu ‘‘Prominence’’ high-performance liquid chromatographer (HPLC) (Shimadzu Corporation, Kyoto, Japan) equipped with an RF-10A XL fluorescence detector. Chromatographic conditions can be found elsewhere [23]. Standard curves of γ- and α-tocopherol (Sigma-Aldrich, Steinheim, Germany) were used for quantifying. Results are expressed as mg of tocopherol per 100 g of dry matter.

Monomeric phenolics and ellagitannins were analyzed by a Shimadzu “Prominence” HPLC apparatus (Shimadzu Corp., Kyoto, Japan) equipped with an RF-10A XL fluorescence detector and an SPD-M20A Diode Array Detector, according to the method described by Arcanjo et al. [16] with some modifications described, as follows. Samples were eluted over a gradient from 7% (0.5% formic acid) to 65% acetonitrile (0.5% formic acid) over 55 min at a rate of 0.5 mL/min. Prior to analysis, fruit extract was filtered using a polyvinylidene difluoride (PVDF) 0.45 μm filter (Agilent) and the injection volume was 2 μL. Based on spectral identification, phenolics were quantified in five subclasses: hydroxybenzoic acids (as gallic acid equivalents, 280 nm), hydroxycinnamic acids (as caffeic acid equivalents, 320 nm), flavonols (as quercetin equivalents, 365 nm), anthocyanins (as delphinidin chloride equivalents, 520 nm) and ellagitannins (as ellagic acid equivalents, 367 nm). Total procyanidin content was quantified by means of acid-catalyzed cleavage reaction in the presence of excess phloroglucinol in accordance with the method described by Arcanjo et al. [16]. Phloroglucinolysis products were identified by comparing their ultraviolet (UV)–visible spectra and retention time with those from standards. A fluorescence detector (λex = 280 nm, λem = 322 nm) was employed simultaneously to improve the identification procedure. Total procyanidins were determined as the sum of the quantified subunits.

### 2.3. In Vitro Bioactivities of Fruit Extracts

The 2,2-diphenyl-1-picrylhydrazyl (DPPH) assay was used to assess in vitro antiradical activity of the fruit extracts. An aliquot of 0.033 mL of each diluted extract (1:50) was mixed with 2 mL DPPH solution (0.06 mM) in methanol. The mixture was vortexed and allowed to stand in the dark and at room temperature for 6 min, and the absorbance at 517 nm was directly measured. A Trolox standard curve (ranging from 0.25 to 2 mM) in 80% methanol was employed. The results were expressed as μM Trolox equivalent antioxidant capacity per g dry matter.

The 2,2′-azino-bis(3-ethylbenzthiazoline-6-sulphonic acid) (ABTS˙⁺) radical cation method described by Re et al. (1999) was also performed with some modifications as follows. The ABTS˙⁺ solution was diluted with ethanol to obtain an absorbance of 0.70 ± 0.04 at 734 nm. Ten μL of each diluted extract (1:50) was added to 1000 μL of ABTS˙⁺ solution and vortexed vigorously. The reaction mixture was allowed to stand in the dark and at room temperature for 6 min and the absorbance at 734 nm was immediately recorded. The absorbance of the reaction samples was compared to that of the Trolox standard curve previously described and the results were calculated as TEAC and expressed as μM Trolox equivalents per g dry matter.

The antioxidant capacity assay was carried out using the CUPRAC method as described by Apak et al. [24] as follows. Fruit extracts were diluted (1:100 in ethanol) and 0.1 mL were mixed with 1 mL CuCl_2_ 10^−2^ M, 1 mL neocuproine solution 7.5 × 10^−3^ M in ethanol, 1 mL of water and 1 mL ammonium acetate buffer at pH 7.0. The mixture was allowed to stand for 30 min in the dark and at room temperature and the absorbance was subsequently measured at 450 nm against a blank. Results were calculated as TEAC and expressed as μM Trolox equivalents per g dry matter.

The in vitro antioxidant activity of fruit extracts against meat protein oxidation was assessed following a modified procedure of that described by Utrera and Estévez [25]. Myofibrillar proteins (5 mg/mL) extracted from porcine muscle was subjected to an in vitro radical-generating system (25 μM FeCl_3_ and 2.5 mM hydrogen peroxide, 37 °C during 24 h and constant stirring). Aliquots of the fruit extracts (200 ppm of GAE) were added to complete five in vitro oxidation systems (including a control group with no fruit extract) prepared in triplicate. Upon completion of the oxidation assay, samples were analyzed for protein carbonyls using the dinitrophenylhydrazine method described by Armenteros et al. [18]. Results were expressed as percent inhibition of protein carbonyl formation using the following formula: 100 × [(C − T)/C], where T and C are the protein carbonyl values of the treated (T) and control (C) samples, respectively.

### 2.4. Preparation of Sprayable Extracts

The sprayable water extracts were produced from each fruit to reach 2000 ppm of GAE. The concentration of the extracts in TPC was selected based on the earliest studies to warrant the desired bioactivity. These extracts (15 mL) were dispensed in 25 mL glass spray bottles.

### 2.5. Experimental Setting and Meat Processing and Sampling

Cutlets were dispensed in polypropylene (PP) trays (Sarabia Plastics, Spain) (130 mm × 160 mm × 50 mm) (0.90 mm thick) and wrapped using polyester/PLPMC film (Wipack, Hamburg, Germany) with an oxygen permeability of 114 cm^3^ m^−2^ day^−1^ bar^−1^ at 25 °C and water transmission rate of 10 g m^−2^ day^−1^ at 38 °C 90 % RH. Cutlets were randomly divided into six types of trays depending on the packaging strategy and the addition of 4 different types of fruit extracts, namely, Vacuum-C (cutlets packed in a vacuum package and no added extract), MAP-C (cutlets packed in a High-Ox-MAP and no added extract), MAP-QI (cutlets packed in a High-Ox-MAP and treated with QI extract), MAP-CM (cutlets packed in a High-Ox-MAP and treated with CM extract), MAP-AU (cutlets packed in a High-Ox-MAP and treated with AU extract) and MAP-RC (cutlets packed in a High-Ox-MAP and treated with RC extract).

For each of these six experimental groups, three additional groups of samples were considered depending on the technological treatment applied to cutlets completing a 6 × 3 factorial design. The technological treatments were as follows: fresh (uncooked) samples (sampled at day 1 after slaughter), refrigerated (10 days at 4 °C under simulated retail display conditions under white fluorescent light [1620 lux]) and cooked (subjected to a roasting procedure at 180 °C for 10 min subsequent to the aforementioned refrigerated storage). Each tray contained 2 cutlets and the whole assay was prepared in duplicate. Each type of sample (6 treatments) and at each sampling time (3 processing stages) consisted of 4 experimental units (*n* = 4) totaling 6 × 3 × 4= 72 lamb cutlets.

Fruit extracts were sprayed on the surfaces of cutlets so that 2 g extract/m^2^ of meat was applied on each side. Samples were allowed to dry at room temperature for 20 min. The concentration of gases in the modified atmosphere package (MAP) was as follows: 50% O_2_, 30% CO_2_ and 20% N_2_. The packaging was carried out using thermo-sealing equipment (Smart 500, ULMA, Oñati, Spain). The concentration of gases in the atmosphere was decided considering results from a previous study [17]. Afterwards, samples were chilled for 10 days at 4 °C under replicated retail display settings.

At sampling times, cutlets were analyzed for thiobarbituric acid reactive substances (TBARS), hexanal, free thiols and α-amino adipic semialdehyde (AAS) content. Fresh and refrigerated samples were analyzed for consumers’ purchasing intent. Cooked samples were assessed for instrumental texture.

### 2.6. Analytical Procedures on Lamb Cutlets

#### 2.6.1. Determination of TBARS Numbers

Malondialdehyde (MDA) and other TBARS were quantified using the method described by Ganhão et al. [26] with slight adjustments. Five grams of lean were homogenized with 15 mL perchloric acid (3.86%) and 0.5 mL BHT (4.2% in ethanol). An ice bath was used to minimize the occurrence of further lipid oxidation during TBARS extraction. The slurry was filtered and centrifuged (600 g for 4 min) and 2 mL aliquots were mixed with 2 mL thiobarbituric acid (0.02 M) in test tubes. After being heated in a boiling water bath (100 °C) for 45 min, the absorbance was measured at 532 nm. For quantitation purposes, a standard curve was used using a 1,1,3,3-tetraethoxypropane (TEP) solution in 3.86% perchloric acid. Results were expressed as mg MDA per kg of meat.

#### 2.6.2. Analysis of Hexanal

Hexanal content was analyzed by gas chromatography/mass spectrometry (GC/MS) following the method described by Estévez et al. [27] with modifications. Analyses were run on Agilent 6890 gas chromatograph equipped with an Agilent Technologies 7697A static headspace sampler (Agilent Technologies, Wilmington, DE, USA) and couple to Agilent Technologies 5975 mass detector. The headspace sampler was connected to the split/splitless port of GC. One gram of minced sample and a 20 mL headspace vials (Restek, Bellefonte, PA, USA) were used in all experiments. The samples were incubated for 30 min at an oven temperature of 40 °C and the temperature of the transfer line connecting the sampler and GC column was 80 °C. The separation of volatiles was performed on a DB-1 column (60 m × 0.250 mm × 1.0 μm) by Agilent Technologies, operating at 6 PSI of column head pressure, resulting in a flow of 1.3 mL min^−1^ at 40 °C. Oven program was: 40 °C for 10 min, 5 °C min^−1^ to 200 °C, 15 C min^−1^ to 250 °C, and held 250 °C for 10 min. The mass spectra were obtained using a mass selective detector by electronic impact at 70 eV, multiplier voltage was 1756 V and data was collected at 1 scan s^−1^ over the m/z range 30–500. Hexanal was positively identified by comparing their mass spectra with those of the standard compounds (Sigma, St. Louis, MO, USA) run under the same conditions. Results from the volatiles analysis are given in area units (AU).

#### 2.6.3. Quantification of α-amino Adipic Semialdehyde (AAS)

The method optimized by Utrera et al. [28] was followed with slight modifications. Five hundred μL of a homogenate prepared from 1 g of lamb cutlet and 10 mL of 2-(N-morpholino) ethanesulfonic acid (MES) buffer pH 6.0, were mixed with 10% (*v*/*v*) trichloroacetic acid (TCA) solution. The mixture was vortexed and subsequently centrifuged at 600 *g* for 5 min at 4 °C. Upon removal of the supernatants, pellets were incubated with 0.5 mL 250 mM MES buffer pH 6.0 containing 1 mM diethylenetriaminepentaacetic acid (DTPA), 0.5 mL 50 mM ABA in 250 mM MES buffer pH 6.0 and 0.25 mL 100 mM NaBH_3_CN in 250 mM MES buffer pH 6.0. After vortexing, samples were incubated in an oven at 37 °C for 90 min. The samples were stirred every 15 min. The samples were then treated with 50% TCA (*v*/*v*) and centrifuged at 1200 *g* for 10 min. The pellets were washed twice with 10% TCA and diethyl ether-ethanol (1:1). Lastly, the hydrolysis of the pellets was carried out with 6N HCl at 110 °C for 18 h. A centrifugal evaporator was employed for drying the hydrolysates *in vacuo*. The remains were redissolved in 200 µL of milliQ water and then filtered through hydrophilic polypropylene GH Polypro (GHP) syringe filters (0.45 μm pore size, Pall Corporation, USA) for HPLC analysis.

A Shimadzu “Prominence” HPLC apparatus (Shimadzu Corporation, Kyoto, Japan), equipped with an RF-10A XL fluorescence detector (FLD) was used. One μL of the protein hydrolysates was injected and AAS-ABA was eluted in a Cosmosil 5C_18_-AR-II RP-HPLC column (5 µm, 150 × 4.6 mm) equipped with a guard column (10 × 4.6 mm) packed with the same material. Detailed chromatographic conditions can be found elsewhere [28]. Identification of AAS was carried out by comparing the retention time with that from the standard compound. AAS was quantified by plotting the chromatographic areas against an ABA standard curve (0.1 to 0.5 mM). Results were expressed as nmol of AAS per mg of protein.

#### 2.6.4. Analysis of Protein Thiols

The analysis of free thiols was carried in accordance with the method described by Lund et al. [7] with minor modifications. Five hundred μL of a homogenate prepared from 1 g of lamb cutlet and 10 mL of 2-(N-morpholino) ethanesulfonic acid (MES) buffer pH 6.0, were dispensed in 2 mL microtubes was washed twice with PBS and ethanol:ethyl acetate (1:1) and treated with cold 10% (*v*/*v*) trichloroacetic acid (TCA). The pellet was resuspended in 250 µL of guanidine hydrochloride and added to the cuvette in a final volume of 1250 µL of guanidine hydrochloride. Absorbance was measured at 324 nm (Hitachi spectrophotometer, Tokyo, Japan), pre- and post-addition of 250 µL of 4,4,4′-Dipyridyl disulfide (DPS) in 12 mM HCl. The results were expressed as nmol of free thiol groups per mg of sample.

#### 2.6.5. Instrumental Hardness

Texture profile analysis (TPA) was performed at room temperature with a Texture Analyzer TA-XT2i (Stable Micro Systems, Surrey, UK). Three cubed samples (1 cm^3^) were taken from the middle of the lamb loin sample and subsequently subjected to a two-cycle compression test. The samples were compressed to 50% of their original weight with a cylindrical probe of 5 cm diameter and a cross-head speed of 5 mm/s. Hardness (N) was calculated as a maximum force applied on the first compression peak. All analyses were performed in triplicate.

#### 2.6.6. Purchase Intention

Sixty consumers (recruited at the University of Extremadura campus, Cáceres, Spain) were asked to express their willingness to purchase the lamb cutlets from the present study. Participants, aged between 25 and 65, identified themselves as regular purchasers and consumers of lamb cutlets. Trays corresponding to fresh and refrigerated lamb cutlets were shown to evaluators who indicated their willingness to purchase the product. Additionally, consumers were asked to justify their decision by providing reasons. Results are expressed as proportion of consumers willing to purchase a particular sample at a particular sampling day.

#### 2.6.7. Statistical Analysis

Sprayable fruit extracts (main factor) were applied independently to each cutlet and packed in pairs. Therefore, each cutlet was regarded as an experimental unit and the entire sample processing (preparation of extracts, spraying process, packaging, chilled storage and cooking) was replicated twice totaling 12 cutlets per group of samples. A random sampling of four cutlets per treatment was carried out at each of the three processing stages (fresh, refrigerated and cooked). A one-way Analysis of Variance (ANOVA) was applied to data from the assessment of samples at each processing stage. Whenever significant differences (*p* < 0.05) were found, Tukey’s posthoc tests were also carried out. The effect of the processing stages was studied using the General Linear Model with repeated measures. In the mixed statistical model, each specific cutlet was regarded as a random effect, while the application of the fruit extract, the processing stage and the interaction between application of fruit × processing stage were considered as fixed effects. Once again, Tukey’s tests were applied as a posthoc statistical method. To assess the consumers’ responses to the questions regarding their preference for samples, chi-square tests were applied to the binary data (Yes/No). The SPSS program (v. 18.0) was employed for the statistical analysis and the level of statistical significance was set at *p* < 0.05.

## 3. Results and Discussion

### 3.1. Characterization of Fruit Extracts

The concentration of bioactive compounds in the extracts elaborated from the four fruits under study is shown in Table 1. All fruits were found to contain large levels of total phenolics as compared to data reported by well-recognized review articles [29]. Among the four fruits, CM was found to contain the highest TFC followed by AU, RC and QI. In regards to specific phenolics, CM was particularly rich in procyanidins (PC) and also had higher concentrations of flavonols (FV) and hydroxycinnamic acids (HCA) than the other group of samples. Conversely, AU had the lowest concentration of procyanidins but contained the highest levels of hydroxybenzoic acids (HBA), together with QI. RC was particularly rich in procyanidins and contained assorted amounts of hydroxycinnamic acids, flavonols and ascorbic acid. QI was found to be the only fruit to contain ellagitannins (ET) and variable amounts of procyanidins, hydroxybenzoic acids and flavonols. CM was also identified as the best source of tocopherols, followed by AU and RC, and eventually by QI, which had the lowest concentration of this bioactive compound. Finally, AU and RC were found to be natural sources of ascorbic acid while CM and QI had significantly lower concentrations. Overall, these results are consistent with data from previous studies. Ganhão et al. [30] reported that the ethanolic extracts from CM had significantly higher TPC than other fruits, including RC and AU. According to those authors, procyanidins were the most abundant phenolic components in CM, which is consistent with the present results. The phenolic profiles of RC and AU described by Ganhão et al. [30], are also in line with those reported here. In regards to QI, the TPC content reported in this study (~2000 mg GAE/100 fruit) is considerably higher than that found by Cantos et al. [31] (~200 mg GAE/100 fruit) or Ferreira et al. [32] (~900 mg GAE/100 fruit) which can be attributed to the optimization of the extraction conditions applied in the present study. QI is commonly known to be a natural source of tocopherols [33]. Yet, the present results indicate that berries such as CM, AU and RC contain larger concentrations than QI of this potent antioxidant.

The in vitro antioxidant activity of the fruits can be attributed to the bioactive compounds aforementioned. Among fruits, AU was found to display the highest antioxidant activity in the DPPH, ABTS and CUPRAC assays, probably due to the well-balanced combination of TPC, tocopherols and ascorbic acid. Fruits with larger TPC such as CM lacked ascorbic acid and the other two fruits under study (QI and RC) had lower concentrations of some of the bioactive components than CM. In the study performed by Ganhão et al. [30], CM displayed the most intense in vitro antioxidant activity against the DPPH and ABTS radicals among a group of fruits, in which RC and AU were included. These results emphasize that the bioactivity of the so-called “natural antioxidants” do not only depend on quantity but on the timely interaction of bioactive compounds. In this regard, the synergy and regeneration mechanisms between tocopherols, ascorbic acid and phenolics are well documented [34]. AU had the highest concentrations of the two former and considerable amounts of the latter. The percent inhibition against the in vitro carbonylation of myofibrillar proteins revealed the mechanistic differences between the classic antioxidant in vitro assays (DPPH, ABTS, CUPRAC) and protein oxidation mechanisms. The impact of antioxidant compounds on the redox state of proteins largely depends on the molecular interactions established between the bioactive compounds and protein residues [35]. This was reflected in the results from the present study as the fruit with higher TPC (CM) had the lower ability to inhibit protein carbonylation. PC are already proposed to be the main contributors to the potent antioxidant activity of RC against protein oxidation in muscle foods [18,36]. In regards to QI and AU, the protection against protein carbonylation can be attributed to HBA, which have also been found to display specific molecular mechanisms to counteract protein oxidation in vitro, such as antiradical and metal chelating activities [25]. The results from the in vitro studies show the indisputable potential of the four fruits to be used as inhibitors of oxidative reactions in complex food systems.

### 3.2. Fruit Extracts as Inhibitors of Lipid Oxidation in Lamb Cutlets

**Figure 1:**
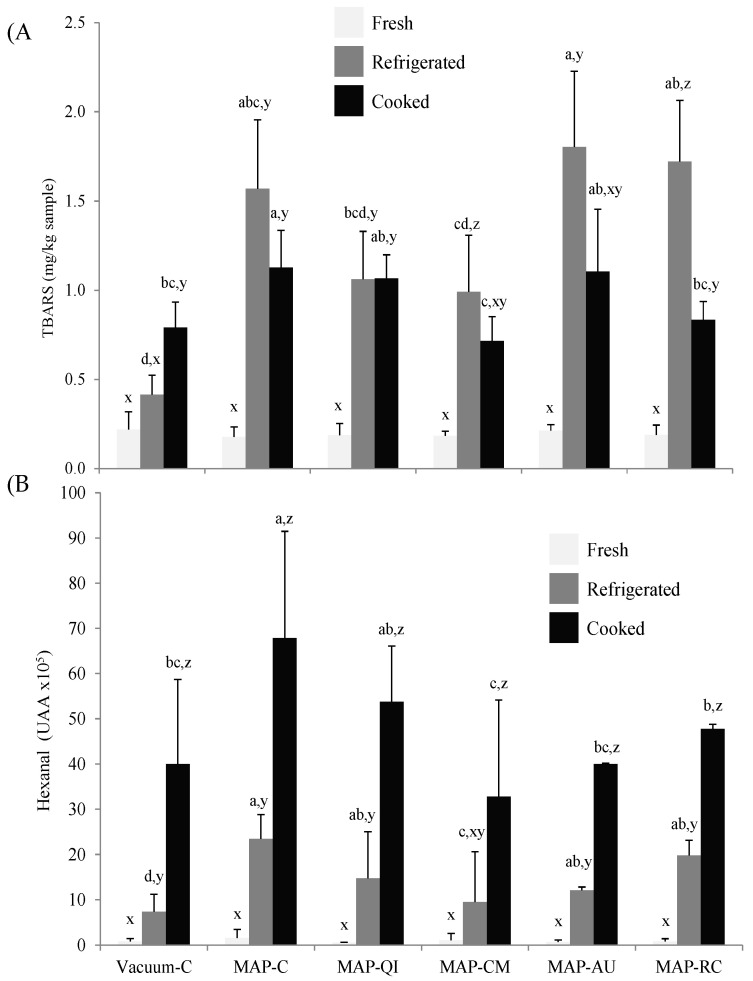
Lipid oxidation markers, thiobarbituric acid reactive substances (TBARS) (**A**) and hexanal counts (**B**) in fresh, refrigerated and cooked lamb cutlets. Sample description: Vacuum-C (cutlets packed in a vacuum package and no added extract), MAP-C (cutlets packed in a high-oxygen packaging atmosphere (High-ox-MAP) and no added extract), MAP-QI (cutlets packed in High-Ox-MAP and treated with QI extract), MAP-CM (cutlets packed in a High-Ox-MAP and treated with CM extract), MAP-AU (cutlets packed in a High-Ox-MAP and treated with AU extract) and MAP-RC (cutlets packed in a High-Ox-MAP and treated with RC extract). ^a–d^ Different letters on top of bars denote significant differences between treatments within a processing stage (Fresh, refrigerated or cooked). ^x–z^ Different letters on top of bars denote significant differences between processing stages within a particular treatment.

The extent of lipid oxidation in fresh, refrigerated and cooked lamb cutlets was assessed by means of the detection of TBARS (Figure 1A) and hexanal (Figure 1B). Both lipid oxidation markers increased during the refrigerated storage of all groups of samples (except TBARS in Vacuum-C), reflecting the occurrence of lipid oxidation during the simulated retail display. Yet, the progress of each of these markers during the subsequent cooking procedure diverged. The concentration of TBARS tended to decrease in all cooked samples and such decrease was statistically significant in MAP-RC. In Vacuum-C, conversely, the concentration of TBARS increased in cooked samples. These results may be caused by the involvement of malondialdehyde and other TBARS in further reactions, which is stimulated by high temperatures during cooking. These reactions include the attachment of lipid-derived carbonyls to proteins via covalent linkages [37]. Similar results were reported in previous studies aimed to assess lipid oxidation in cooked meats subsequent to refrigerated storage [32,38]. It is worth noting that such a decrease is particularly remarkable in samples with high TBARS levels. Therefore, it is reasonable to consider that a variable proportion of the TBARS formed and accumulated in meat during chilled storage is reacting and hence, depleted during the subsequent cooking. Conversely, the hexanal counts increased gradually and significantly at each of the processing stages, with the more intense increase being detected upon cooking. In this regard, hexanal may be regarded as a more reliable indicator of lipid oxidation as it precisely reflects the extent of lipid oxidative damage caused by diverse technological processes [26,27,38]. To similar conclusions came other researchers by observing the erratic evolution of TBARS during severe and prolonged processing [32,38,39].

The effect of the packaging strategy on lipid oxidation was remarkable and, in this case, both TBARS and hexanal measurements agreed in showing significantly higher oxidation levels in refrigerated and cooked meats packed in MAP-C than in those packed in Vacuum-C. The high concentration of molecular oxygen in MAP is known to facilitate the onset of a pro-oxidative environment that leads to increased lipid oxidation rates [11,12]. The pro-oxidant effect of High-Ox-MAP on lipid oxidation has been reported for pork steaks [12], chicken drumsticks [40], beef muscles [41] and lamb cutlets [17], among others. The application of the sprayed fruit extracts from CM (MAP-CM) and RC (MAP-RC) caused a significant reduction of TBARS in cooked lamb cutlets as compared with the control counterparts (MAP-C). In regards to hexanal counts, antioxidant protection was significantly achieved by CM in refrigerated lamb cutlets and by CM, AU and RC in cooked samples. The effectiveness of these fruits as antioxidants has been reported before in frankfurters subjected to chilled storage (RC, AU) [42], chilled and cooked beef patties (RC) [38] and ready-to-eat porcine patties (CM) [43]. Armenteros et al. [18] reported significant antioxidant effects of injected extracts from RC on cooked hams refrigerated for 150 days. As an original contribution, the present study confirms the effectiveness of sprayable extracts from CM, AU and RC in reducing lipid oxidation, as assessed by hexanal formation, in cooked lamb cutlets. The higher concentration of phenolic compounds and tocopherols in CM could be responsible for the more intense and versatile antioxidant effects of this fruit in the meat samples as compared to the other fruits under evaluation. QI extracts were unable to inhibit lipid oxidation in lamb cutlets subjected to High-Ox-MAP and subsequent cooking. These results contrast with those reported by Ferreira et al. [32] who observed a high efficient antioxidant effect of QI extracts in chicken patties subjected to chilled storage, cooking and subsequent reheating. It is worth noting that in the study by Ferreira et al. [32], QI extract was thoroughly mixed with ground chicken meat and hence, the accessibility of bioactive compounds to lipids would have been facilitated. According to the present results, the remarkable antioxidant potential of QI emphasized in previous papers [32,33] may not be applicable when sprayed on the surface if intact meat pieces.

The intake of MDA and other TBARS are known to have a negative impact on human health [44]. Furthermore, given the direct involvement of lipid oxidation products on sensory traits (rancidity, discoloration), it is plausible to consider that the benefits of the antioxidant protection of some of the fruits under study would be reflected in a more positive sensory, nutritional and health profile of lamb cutlets.

### 3.3. Fruit Extracts as Inhibitors of Protein Oxidation in Lamb Cutlets

**Figure 2:**
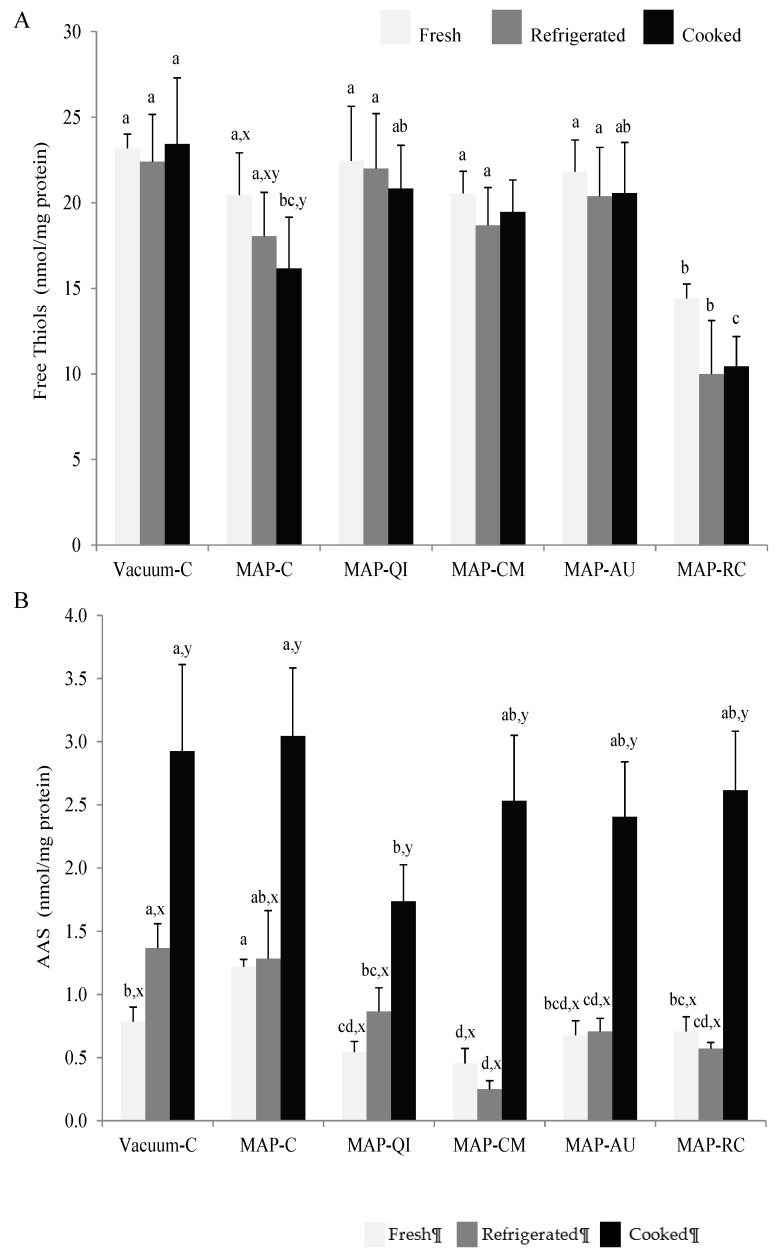
Concentration (nmol/mg protein) of protein thiols (**A**) protein oxidation marker, α-amino adipic semialdehyde (AAS) (**B**) (mean ± standard deviation), in fresh, refrigerated and cooked lamb cutlets. Sample description: Vacuum-C (cutlets packed in a vacuum package and no added extract), MAP-C (cutlets packed in a High-Ox-MAP and no added extract), MAP-QI (cutlets packed in High-Ox-MAP and treated with QI extract), MAP-CM (cutlets packed in a High-Ox-MAP and treated with CM extract), MAP-AU (cutlets packed in a High-Ox-MAP and treated with AU extract) and MAP-RC (cutlets packed in a High-Ox-MAP and treated with RC extract). ^a–d^ Different letters on top of bars denote significant differences between treatments within a processing stage (Fresh, refrigerated or cooked). ^x–z^ Different letters on top of bars denote significant differences between processing stages within a particular treatment.

In the present study, the oxidative damage to proteins in lamb cutlets was assessed by means of thiols depletion (Figure 2A) and accretion of the protein carbonyl, AAS (Figure 2B). Sulphur-containing amino acids in meat systems are sensitive to the pro-oxidant action of ROS and easily undergo oxidative degradation [1]. In fact, the loss of free thiols in muscle foods has been profusely used as an indicator of early protein oxidation changes [1]. The level of thiols remained unchanged during chilled storage and subsequent cooking of lamb cutlets subjected to the vacuum package. This result illustrates the benefit of oxygen exclusion in terms of protection against protein oxidation in meat systems. Conversely, the High-Ox-MAP led to an ongoing depletion of thiols during the processing stages that reached a significant extent in the cooked samples. The effects of High-Ox-MAP vs. vacuum packaging on the oxidation of free thiols during meat storage and processing was already reported by Jongberg et al. [4] and Fu et al. [45], among others. The sprayable extracts from QI, CM and AU protected protein thiols against oxidation as no thiol depletion was observed for MAP-QI, MAP-CM and MAP-AU samples during any of the three processing stages. In relation to existing literature, several authors have described the protective effect of phenolic-rich plant extracts such as those from rosemary [46] and white grape [47] on protein thiols from meat patties subjected to High-Ox-MAP. It worth noticing that unlike the aforementioned studies, the antioxidant effect of sprayable extracts was proven in an intact meat product.

RC had the opposite effect as the application of the extract from this fruit led to an immediate decrease of thiols in fresh MAP-RC samples. As for MAP-C counterparts, processing of MAP-RC samples caused a depletion of thiols that appeared to be significant at the cooking stage. Therefore, the antioxidant protection displayed by RC extracts against lipid oxidation in cooked lamb cutlets, contrast with the apparent pro-oxidant effect exerted on protein thiols. The divergence between the effects of phenolic-rich extracts on lipid and protein oxidation illustrates the differences between both phenomena in terms of chemical mechanisms and pathways. It is known that certain polyphenols are able to form stable covalent linkages with cysteinyl thiols in proteins [21,47] and procyanidins in particular, have been found to display high affinity rates [21,47]. Though this mechanism could be regarded as a pro-oxidant action as thiols are depleted, the location of the attached phenolic on the surface of the protein could in fact facilitate the protection of the latter against further oxidation threats [1]. The pro-oxidant effects of particular phenolics, such as those found in RC, have also been documented [25]. Nieto et al. [48] reported a severe pro-oxidant effect of a phenolic-rich garlic extract manifested as a dramatic loss of protein thiols in refrigerated pork patties.

The effect of the packaging strategy and application of sprayable extracts from four fruits on protein oxidation was also assessed by means of protein carbonylation. In particular, the specific oxidation product from lysine, AAS, was detected and quantified in lamb cutlets. AAS is formed as a result of the oxidative deamination of free and/or protein-bound lysine residues in the presence of ROS [37]. Interestingly, the refrigerated storage did not affect the concentration of AAS in most samples. In fact, the concentration of AAS was similar in Vacuum-C and MAP-C after the refrigerated storage and the subsequent cooking, indicating that oxygen concentration did not promote protein carbonylation. This is reasonable as the formation of AAS in proteins can take place in the absence of molecular oxygen, while other pro-oxidant factors such as the occurrence of transition metals (i.e., iron), hydrogen peroxide and ROS, are highly influential [2,37].

Other previous works reported a promoting effect of High-Ox-MAP on meat protein carbonylation [40,45]. Yet, those aforementioned studies employed the unspecific dinitrophenylhydrazine method that, according to a recent report by Estévez et al. [37], could overestimate protein oxidation by accounting for lipid-derived carbonyls. In regards to the effect of the sprayable fruit extracts as compared to control groups, QI and CM reduced AAS formation in fresh cutlets, CM, AU and RC during refrigeration and only QI was able to significantly reduce AAS formation in cooked samples. The protection of meat proteins by CM, AU and RC has been previously reported in ground muscle foods [36,42,43]. The remarkable inhibitory effects on AAS formation displayed by QI contrast with the weak potential showed against lipid oxidation. Once again, these results support the hypothesis that the behavior and redox-effects of plant phenolics on meat systems are variable between lipids and proteins. A detailed analysis of the bioactive components (Table 1) allows hypothesizing on which components from QI could be contributing to this antioxidant effect on meat proteins. Since QI had the lowest concentrations of tocopherols and ascorbic acid, it seems reasonable to find the responsible for the effect on proteins among phenolics. Among the four fruits, QI was the only to contain ellagitannins, a group of phenolics to which a number of bioactivities have been ascribed [22,29]. Though the interaction mechanisms and redox effects of ellagitannins on meat proteins have been scarcely studied, Viljanen et al. [49] documented antioxidant effects of these phenolics on dairy proteins oxidized in vitro. The molecular basis of the effect of ellagitannins on meat proteins is worth investigating as QI and other fruits rich in such a group of phenolics may be suitable candidates to protect against protein oxidation in muscle foods. In fact, a recent study by Ferreira et al. [32] reported intense antioxidant effects of QI extracts on protein oxidation in ultra-processed chicken patties, including significant inhibition of protein carbonylation.

**Figure 3:**
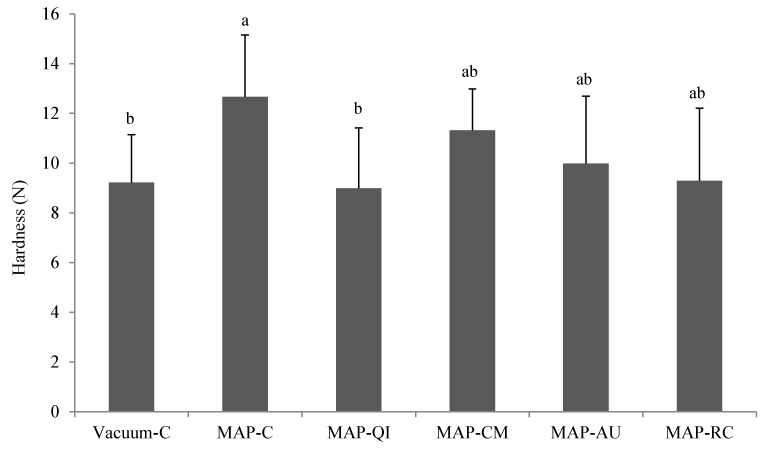
Instrumental hardness (N) of cooked lamb cutlets (mean ± standard deviation). Sample description: Vacuum-C (cutlets packed in a vacuum package and no added extract), MAP-C (cutlets packed in a High-Ox-MAP and no added extract), MAP-QI (cutlets packed in High-Ox-MAP and treated with QI extract), MAP-CM (cutlets packed in a High-Ox-MAP and treated with CM extract), MAP-AU (cutlets packed in a High-Ox-MAP and treated with AU extract) and MAP-RC (cutlets packed in a High-Ox-MAP and treated with RC extract). ^a,b^ Different letters on top of bars denote significant differences between treatments.

The protection against protein oxidation leads to a number of benefits in muscle foods and includes better protein quality and protein digestibility [2] and protection against the formation of potentially harmful compounds [4]. Furthermore, protein oxidation has also been found to be responsible for the impairment of quality traits in muscle foods such as texture. Based on findings by Lund et al. [7], among others, the occurrence of protein oxidation in meat subjected to High-Ox-MAP leads to increased toughness owing to the formation of protein crosslinks. Though such protein structures were not assessed in the present study, protein carbonyls such as AAS are known to be precursors of protein aggregates [37,50]. In accordance with findings by Lund et al. [7], keeping lamb cutlets in High-Ox-MAP led to increased instrumental hardness as compared to the Vacuum-C counterparts (Figure 3). Among the fruit extracts, only QI was able to significantly counteract the pro-oxidative effect of the MAP on protein oxidation and the increased hardness. The timely consistency between AAS results and instrumental hardness provides strength to the hypothesis that AAS may be involved in the formation of protein crosslinks and the onset of tough textures in meat subjected to High-Ox-MAP. It is worth noting that even though previous studies have reported similar effects of other phenolic-rich plant materials, this is, to our knowledge, the first time that sprayable fruit extract displays such a significant effect on intact lamb meat subjected to High-Ox-MAP.

### 3.4. Impact of Fruit Extracts and Packaging on Consumers’ Purchase Intention

**Figure 4:**
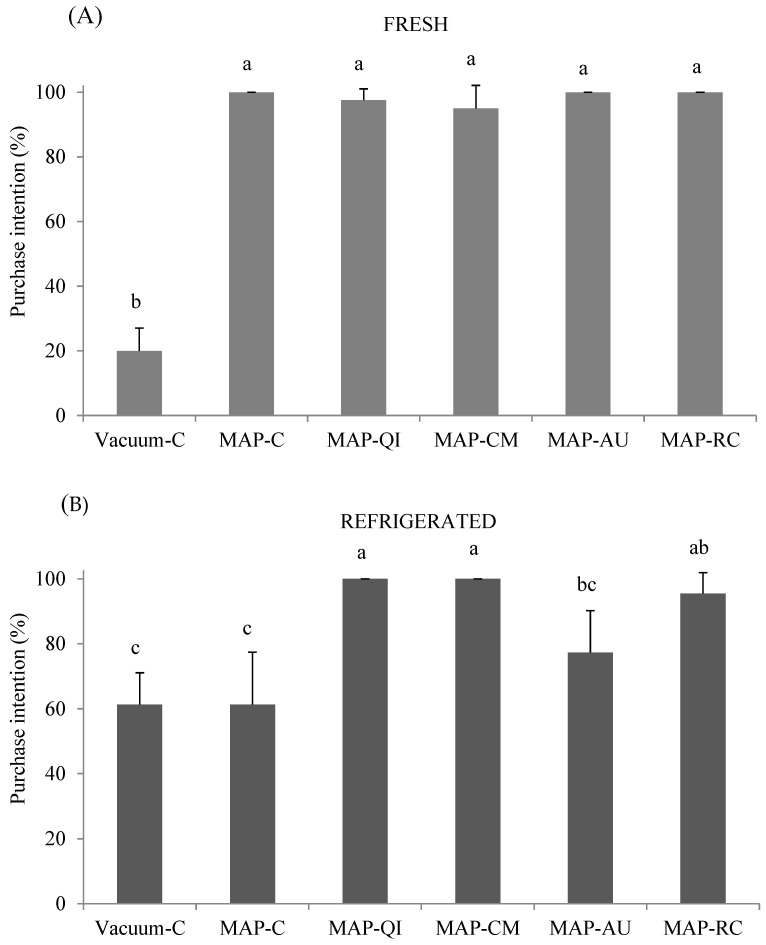
Consumers’ purchase intention (%) after visual assessment of fresh (**A**) and refrigerated (**B**) lamb cutlets (mean ± standard deviation). Sample description: Vacuum-C (cutlets packed in a vacuum package and no added extract), MAP-C (cutlets packed in a High-Ox-MAP and no added extract), MAP-QI (cutlets packed in High-Ox-MAP and treated with QI extract), MAP-CM (cutlets packed in a High-Ox-MAP and treated with CM extract), MAP-AU (cutlets packed in a High-Ox-MAP and treated with AU extract) and MAP-RC (cutlets packed in a High-Ox-MAP and treated with RC extract). ^a–c^ Different letters on top of bars denote significant differences between treatments.

In order to assess the extent to which the effects observed for the packaging strategies and fruit extracts could actually have an influence on the final consumer, purchase intention by consumers based on the visual appearance of fresh (Figure 4A) and refrigerated (Figure 4B) lamb cutlets, was performed. At the beginning of the assay, the High-Ox-MAP was clearly preferred by consumers over the vacuum-packaged lamb cutlets. Nearly all consumers expressed their intention to purchase all MAP trays, irrespective of the application of the fruit extract, while only 19% of these consumers expressed their willingness to purchase the vacuum-C counterparts. The formation of oxymyoglobin and the display of bright red color are documented benefits of High-Ox-MAP [11,13] and this may have driven consumers’ preference towards MAP samples.

The differences between types of samples changed considerably after the refrigerated storage. The purchase intention of MAP-C dropped to ~60%, matching the percentage of vacuum-C trays. The impairment of lamb appearance probably due to the combination of microbial spoilage and discoloration (not measured in the present study) could explain these results with the vacuum samples (protected against aerobic bacteria and color changes due to oxygen exclusion) were unaffected by these factors. In a previous study, both discoloration and growth of spoilage bacteria were found to occur during the retail display of lamb cutlets packed in High-Ox-MAP [17]. Compared to the MAP-C, the application of sprayable extracts of QI, CM and RC, significantly improved consumers’ purchase intention based on appearance. It is, therefore, reasonable to consider that such an improvement could have been caused by the antioxidant effects of these extracts. Yet, a potential antimicrobial effect could have also contributed to improving the appearance since the fruits under study have been reported to display such effects in vitro and in complex muscle foods [15,29,31].

## 4. Conclusions

Whereas High-Ox-MAP is commonly used in red meat retail displays, its effects on protein oxidation are noticeable. The application of extracts from fruits such as QI seems to be a feasible and efficient solution to reduce the pro-oxidative effects caused by High-Ox-MAP in red meat. The protective effect of QI on meat proteins is also reflected in decreased hardness and increased consumer purchasing decisions. Yet, the present results reflect that the effectiveness of sprayable fruit extracts on meat protein oxidation highly depends on the content and composition of bioactive compounds. Therefore, a successful practical application of such a strategy requires a previously detailed characterization of the extracts and an understanding of the underlying molecular mechanisms.

## Figures and Tables

**Table 1:** Total phenolic content (TFC), tocopherol, vitamin C content and phenolic profile, and in vitro bioactivities of extracts from assorted fruits, namely, oaknut (*Quercus ilex* subsp. *ballota;* QI), common hawthorn (*Crataegus monogyna* Jacq.; CM), strawberry tree (*Arbutus unedo* L., AU) and rose hips (*Rosa canina* L.; RC).

	QI			CM			AU			RC			*p* ^A^
TFC ^B^	2055c	±	356	4362a	±	113	3480b	±	33	3117b	±	236	0.000
Tocopherol ^C^	0.58c	±	0.09	2.09a	±	0.36	1.51b	±	0.05	1.25b	±	0.11	0.000
Ascorbic acid ^D^	0.05c	±	0.05	0.12c	±	0.07	6.47a	±	0.48	4.79b	±	0.06	0.000
Phenolic profile ^C^													
HBA	41.8a	±	4.9	0.8b	±	0.4	40.9a	±	3.8	N.D			0.000
HCA	N.D			38.9a	±	8.1	N.D			19.1b	±	1.7	0.000
FV	1.50c	±	0.34	36.26a	±	0.84	1.00c	±	0.21	9.03b	±	1.08	0.000
AC	N.D			1.39b	±	0.24	2.33a	±	0.20	0.77c	±	0.10	0.000
ET	317	±	31	N.D			N.D			N.D			0.000
PC	904c	±	45	1498b	±	126	315d	±	39	2322a	±	140	0.000
Total	1264c	±	133	1575b	±	168	359d	±	38	2350a	±	36	0.000
Antioxidant activity ^E^													
DPPH	145ab	±	6	232a	±	48	218a	±	64	90b	±	3	0.008
ABTS	198	±	25	153	±	12	204	±	3	201	±	28	0.036
CUPRAC	354b	±	10	385ab	±	20	434a	±	23	431a	±	39	0.012
% Inhibition POX ^F^	67.5a	±	0.3	31.4b	±	3.3	68.6a	±	0.4	73.6a	±	7.6	0.000

HBA: hydroxybenzoic acids; HCA: hydroxycinnamic acids; FV: flavonols; AC: anthocyanins; ET: ellagitannins; PC: procyanidins; DPPH: 2,2-Diphenyl-1-picrylhydrazyl; ABTS: 2,2′-Azino-bis(3-ethylbenzothiazoline-6-sulfonic acid); CUPRAC: cupric-reducing antioxidant capacity; ND: no detected. ^A^ Significance in ANOVA; Means with different letters in a row denote significant differences between fruits. ^B^ mg gallic acid equivalents (GAE) per 100 g of dry matter. ^C^ mg per 100 g of dry matter. ^D^ mg per g of dry matter. ^E^ μM Trolox equivalents per g dry matter. ^F^ Percent inhibition against the formation of protein carbonyls in myofibrillar proteins in vitro oxidation assay.
